# Editorial: Global excellence in neurogenesis: Europe

**DOI:** 10.3389/fnins.2023.1335276

**Published:** 2023-11-27

**Authors:** Ángela Fontán-Lozano

**Affiliations:** ^1^Department of Physiology, School of Biology, Universidad de Sevilla, Seville, Spain; ^2^Instituto de Biomedicina de Sevilla, Hospital Universitario Virgen Del Rocío, Consejo Superior de Investigaciones Científicas, Universidad de Sevilla, Seville, Spain

**Keywords:** neurogenesis, adult neurogenesis, hippocampus, neural stem cell physiology, cell differentiation, circuit integration

We are highlighting the latest advances in neurogenesis worldwide and reflecting on the future challenges faced by transboundary researchers. This Research Topic address the advancement of neurogenesis research in Europe, and presents some examples of recent work regarding several aspects of this investigation field, which can be assigned to the following subthemes: phylogeny, normal/disease physiology, and new avenues to analyse neurogenesis. To put into context these subthemes, in this Editorial, we summarize the main challenges being addressed nowadays in the field.

## Phylogeny

Vertebrates exhibit varying degrees of adult neurogenesis. Along the phylogeny, there seems to be an apparent reduction in the extent and number of nervous system locations where it takes place (Kempermann, 2015; Terreros-Roncal et al., 2023). An exciting subfield of research on adult neurogenesis phylogeny is the analysis of whether it is evolutionarily conserved and a taxonomically widespread phenomenon, due to the relevance of characterizing *de novo* neurogenesis and regeneration. This aspect is addressed by González-Granero et al. in this Research Topic, conducting an ultrastructural characterization of the neural precursors present in the ventricular zone of a lizard. The authors show the identity and distribution of neural stem cells and their proliferative activity, neuroblast migration routes at the time of differentiation in the olfactory bulb, and neuronal maturation in the medial cortex of Podarcis liolepis. Given varying neurogenesis extent and duration, it's crucial to consider accumulated data on diverse regulatory mechanisms at cellular and molecular levels.

## Cellular and molecular regulation of adult neurogenesis

Adult neurogenesis regulation has been extensively studied, providing comprehensive insights into neural stem cell quiescence, cell cycle entry, molecular mechanisms, and various aspects of newborn neuron life, such as self-renewal, differentiation, and survival (Llorens-Martín et al., 2009; Egeland et al., 2015; Kempermann et al., 2015; Urbá et al., 2019; Denoth-Lippuner and Jessberger, 2021). One of the most intriguing aspects of this regulation is how newborn neurons grow and extend their processes to get connected in an already established, mature circuit.

Cell differentiation and circuit integration have been largely described in the last decade and a half, accumulating very detailed descriptions about how it works, including synapse formation and functional integration (Toni and Schinder, 2016; Mira and Morante, 2020; Denoth-Lippuner and Jessberger, 2021; Ribeiro and Xapelli, 2021).

## Function of adult neurogenesis

The role of adult neurogenesis was indirectly investigated for years through the analysis of the behavior of mice models after genetic, pharmacological, or interventional increases and decreases of newborn cell numbers, reviewed by Llorens-Martín et al. (2009). After the publication of the first mouse models genetically designed to address directly a lack or a substantial decrease of adult hippocampal neurogenesis, it was clear the direct involvement of adult neurogenesis in the behavior and specifically both in learning and memory and in specific mood traits (Abrous et al., 2005, 2022; Dupret et al., 2007; Cameron and Glover, 2015). This bulk evidence clearly reveals the relevance of investigating the physiology of these cells both in normal and disease conditions.

## Normal/disease physiology

In the last three decades, an analysis of the physiology of adult neurogenesis in healthy and diseased organisms has accumulated a huge amount of information. Thanks to this evidence we know today that adult neurogenesis correlates (and in some cases, it has been directly related to the etiopathogenesis) with a number of brain conditions, including neurodegenerative diseases, depression, and fronto-temporal dementia, among others, reviewed by Toda et al. (2019), Berger et al. (2020), and Babcock et al. (2021). This aspect of adult neurogenesis has special relevance considering that it has been demonstrated to exist in humans and there are decreased newborn cell numbers and function associated to neurodegeneration-related cognitive decline (Moreno-Jiménez et al., 2021). In this subfield of investigation, Clavijo and Suárez-Martin studied for this Research Topic the rate of proliferation and morphology of neural stem cells, as the first step in the neurogenic process and due to the impact that any alteration can induce on the neurogenic phenomenon.

They analyzed three models of neuronal hyperexcitation: epilepsy induced by different doses of kainic acid, sub-seizure epileptiform activity, and a model of traumatic brain injury, and three models of neuroinflammation: LPS, Poly I:C, IFN-alpha. The authors report a very specific response of these systems, showing the fine-tuning of neural stem cells in response to different interventions. This topic has been widely discussed; see, for example, Encinas and Pineda, 2016.

The authors describe a direct involvement of astrocytes in the specific response, which depends on the type of intervention applied.

Their results reveal clear differences in the hyperexcitation vs. neuroinflammation processes on neural stem cells and, consequently, in adult neurogenesis.

## New frontiers of analyses

Some new approaches have allowed very recent discoveries beyond the classical concepts of adult neurogenesis. In this Research Topic the authors address two fascinating issues: the presence of immature neurons in brain regions not usually described as having adult neurogenesis (cerebral cortex), and the use of novel experimental approaches to the study of neurogenesis, like organoids. Specifically, Coviello et al. analyze the distribution, fine structure, and neurochemical phenotype of the immature neurons in the adult human cerebral cortex, finding interesting results potentially useful for the investigation of some diseases such as epilepsy. The authors report a clear identification of immature cells as neurons, and a characterization of the features of these cells. They also discuss the gaps pending to investigate in this subpopulation of potential “reservoir” young plastic neurons.

In this line, Damianidou et al. review the advantages and limitations of the *in vivo* and *in vitro* models used to investigate the neurodevelopmental disorders and malformations of cortical development, starting from a description of the basic steps of brain development and evolutionary differences among mammals. After thorough descriptions and revision of the literature, they show a detailed picture of the pros and cons (that should include nevertheless ethical issues) of the new models to analyze a functional cerebral cortex in the laboratory, also addressing the limitations of brain organoids.

## Author contributions

ÁF-L: Conceptualization, Writing – original draft, Writing – review & editing.
